# Supplementary material for: Needle‐Plug/Piston‐Based Modular Mesoscopic Design Paradigm Coupled With Microfluidic Device for Point‐of‐Care Pooled Testing
Source: Adv Sci (Weinh). 2024 Sep 13;11(42):2406076. doi: 10.1002/advs.202406076 (PMC11558091; doi:10.1002/advs.202406076)
Supplement: Supplementary file 1 — Supporting Information [file ADVS-11-2406076-s007.pdf]

ADVANCED SCIENCE
Open Access

Supporting Information

for *Adv. Sci.*, DOI 10.1002/advs.202406076

Needle-Plug/Piston-Based Modular Mesoscopic Design Paradigm Coupled With Microfluidic Device for Point-of-Care Pooled Testing

Baobao Lin, Bao Li, Wu Zeng, Yulan Zhao, Huiping Li, Yin Gu and Peng Liu**

Supporting Information

Needle-Plug/Piston-Based Modular Mesoscopic Design Paradigm Coupled with Microfluidic Device for Point-of-Care Pooled Testing

Baobao Lin, Bao Li, Wu Zeng, Yulan Zhao, Huiping Li, Yin Gu, and Peng Liu **

This supplementary file includes the following information:

- Table S1 The sequence of primers used in this study.
- Table S2 Comparison of macro-micro fluid Interfaces in microfluidics.
- Table S3 Comparison of modular design in fluid handling.
- Figure. S1 Key factors affecting barrel diameter.
- Figure. S2 Effect of rubber plug and hollow needle parameters on penetration force.
- Figure. S3 Experimental details of the on-off cycle for reagent injection.
- Figure. S4 Optimization of eluted solution volume.
- Figure. S5 Structure and operation of the nucleic acid extraction cassette.
- Figure. S6 Fluid schematic of the cassette.
- Figure. S7 The modular design of the nucleic acid analyzer.
- Figure. S8 Schematic and performance parameters of the fluid driving module.
- Figure. S9 Schematic and performance parameters of the temperature cycling module.
- Figure. S10 The performance validation of the temperature cycling module.
- Figure. S11 Structure and performance of the fluorescence scanning module.
- Figure. S12 The RNA capture efficiency of the silica membrane.
- Figure. S13 The iDEP system.
- Figure. S14. Fluorescence curve plots from the iDEP system amplification process (1-44).
- Figure. S15. Fluorescence curve plots from the iDEP system amplification process (45-76).
- Figure. S16 Comparison of CT values obtained through iDEP and conventional qPCR method.
- Figure. S17 Force testing apparatus for puncturing rubber with a needle.
- Figure. S18 Photograph of the fluid testing platform.

Table S1 The sequence of primers used in this study.

Primers	Sequence (5'-3')
N-F	GCTTCTGACACAACTGTGTTCAC
N-R	CGGCAGACTTCTCCACAGGAGT
N-P	5'-Cy5-ACCTCAAACAGACACCATGG -BHQ1-3'
ORF1ab-F	ACGGGTTTGCGGTGTAAGTGCAG
ORF1ab-R	AGATGTCAAAAGCCCTGTATACG
ORF1ab-P	5'-FAM-ACACCGTGCGGCACAGGCACTAG-BHQ1-3'
RNase P-F	AGATTTGGACCTGCGAGCG
RNase P-R	GAGCGGCTGTCTCCACAAGT
RNase P-P	5'-VIC-TTCTGACCTGAAGGCTCTGCGCG-BHQ1-3'

Table S2 Comparison of macro-micro fluid interfaces in microfluidics.

Materials	Fluidic Connections	Pressure Compatibility	Dead Volume	Throughput	Integration	Reusability	Operation	Cost	Ref.
Soft（PDMS）	Needle Insertion	100-700Kpa	Low	Low	Hard	Yes	Hard	Low	[1,2]
	Negative pressure adsorption	336 kPa\ 101kPa	Low	High	Hard	Yes	Hard	Low	[3,4]
	plug	≥103 kPa	Medium	Low	Hard	Yes	Medium	Medium	[5]
Rigid (PMMA, PP, PC)	Screw Fastening	630 kPa	Medium	Medium	Hard	Yes	Medium	High	[6]
	O-Ring + Spring	2 MPa	Medium	Low	Hard	Yes	Medium	High	[7]
	Mechanical Lock	200kPa	Medium	Low	Hard	Yes	Medium	High	[8]
	Magnetic	250 kPa+~500 kPa	Medium	Low	Hard	Yes	Medium	High	[9]
	Adhesive Tape	≥1MPa	High	Low	Hard	No	Hard	Low	[10]
	Epoxy Adhesive	≥2MPa	High	Low	Hard	No	Hard	Low	[11]
	Luer Lock	≥2MPa	High	Low	Hard	Yes	Easy	High	[12]
	**Our methods**	**≥2MPa**	**Low**	**High**	**Easy**	**Yes**	**Easy**	**Low**	/

Table S3 Comparison of modular design in fluid handling.

Fluid Handling Range	Modular Design Method	Integration	Programmable Fluid Control	Reagent Storage	Throughput	High-Temperature Tolerance	Ref.
<100μL	Capillary force	Yes	Yes	No	High	No	[13]
	Electrowetting	Yes	Yes	No	High	Yes	[14]
	Pneumatic valves	Yes	Yes	No	High	Yes	[15]
>100uL	Backplate positioning	No	No	No	Low	No	[16-19]
	Coaxial rotation	No	No	No	Low	No	[20,21]
	Magnetic coupling	No	No	No	Low	No	[22]
	Threaded connection	No	No	No	Low	Yes	[23]
	Check valve	Yes	Yes	No	High	No	[24]
	**Our method**	**Yes**	**Yes**	**Yes**	**High**	**Yes**	****

References

1 Christensen, A. M., Chang-Yen, D. A., Gale, B. K. J. J. o. M. & Microengineering. Characterization of interconnects used in PDMS microfluidic systems. **15**, 928 (2005).

2 Hong, J. W., Studer, V., Hang, G., Anderson, W. F. & Quake, S. R. A nanoliter-scale nucleic acid processor with parallel architecture. *Nat Biotechnol* **22**, 435-439, doi:10.1038/nbt951 (2004).

3 Chen, A. & Pan, T. Fit-to-Flow (F2F) interconnects: universal reversible adhesive-free microfluidic adaptors for lab-on-a-chip systems. *Lab Chip* **11**, 727-732, doi:10.1039/c0lc00384k (2011).

4 Cooksey, G. A., Plant, A. L. & Atencia, J. A vacuum manifold for rapid world-to-chip connectivity of complex PDMS microdevices. *Lab Chip* **9**, 1298-1300, doi:10.1039/b820683j (2009).

5 Scott, A., Au, A. K., Vinckenbosch, E. & Folch, A. A microfluidic D-subminiature connector. *Lab Chip* **13**, 2036-2039, doi:10.1039/c3lc50201e (2013).

6 Wilhelm, E., Neumann, C., Duttenhofer, T., Pires, L. & Rapp, B. E. Connecting microfluidic chips using a chemically inert, reversible, multichannel chip-to-world-interface. *Lab Chip* **13**, 4343-4351, doi:10.1039/c3lc50861g (2013).

7 Kortmann, H., Blank, L. M. & Schmid, A. A rapid, reliable, and automatable lab-on-a-chip interface. *Lab Chip* **9**, 1455-1460, doi:10.1039/b820183h (2009).

8 Yang, Z. & Maeda, R. Socket with built-in valves for the interconnection of microfluidic chips to macro constituents. *J Chromatogr A* **1013**, 29-33, doi:10.1016/s0021-9673(03)01125-7 (2003).

9 Atencia, J. *et al.* Magnetic connectors for microfluidic applications. *Lab Chip* **10**, 246-249, doi:10.1039/b913331c (2010).

10 Glavan, A. C. *et al.* Rapid fabrication of pressure-driven open-channel microfluidic devices in omniphobic R(F) paper. *Lab Chip* **13**, 2922-2930, doi:10.1039/c3lc50371b (2013).

11 Pattekar, A. V., Kothare, M. V. J. J. o. M. & Microengineering. Novel microfluidic interconnectors for high temperature and pressure applications. **13**, 337 (2003).

12 Nie, M. & Takeuchi, S. Luer-lock valve: A pre-fabricated pneumatic valve for 3D printed microfluidic automation. *Biomicrofluidics* **14**, 044115, doi:10.1063/5.0020531 (2020).

13 Olanrewaju, A., Beaugrand, M., Yafia, M. & Juncker, D. Capillary microfluidics in microchannels: from microfluidic networks to capillaric circuits. *Lab Chip* **18**, 2323-2347, doi:10.1039/c8lc00458g (2018).

14 Liu, X. *et al.* Electrowetting-based digital microfluidics: Toward a full-functional miniaturized platform for biochemical and biological applications. *TrAC Trends in Analytical Chemistry* **166**, 117153, doi:https://doi.org/10.1016/j.trac.2023.117153 (2023).

15 Shaikh, K. A. *et al.* A modular microfluidic architecture for integrated biochemical analysis. *Proc Natl Acad Sci U S A* **102**, 9745-9750, doi:10.1073/pnas.0504082102 (2005).

16 Owens, C. E. & Hart, A. J. High-precision modular microfluidics by micromilling of interlocking injection-molded blocks. *Lab Chip* **18**, 890-901, doi:10.1039/c7lc00951h (2018).

17 Vittayarukskul, K., Lee, A. P. J. J. o. M. & Microengineering. A truly Lego®-like modular microfluidics platform. **27**, 035004 (2017).

18 Yuen, P. K., Bliss, J. T., Thompson, C. C. & Peterson, R. C. Multidimensional modular microfluidic system. *Lab Chip* **9**, 3303-3305, doi:10.1039/b912295h (2009).

19 Yuen, P. K. J. L. o. a. C. SmartBuild–A truly plug-n-play modular microfluidic system. **8**, 1374-1378 (2008).

20 Bhargava, K. C., Thompson, B. & Malmstadt, N. Discrete elements for 3D microfluidics. *Proc Natl Acad Sci U S A* **111**, 15013-15018, doi:10.1073/pnas.1414764111 (2014).

21 Lai, X. *et al.* A Rubik's microfluidic cube. *Microsyst Nanoeng* **6**, 27, doi:10.1038/s41378-020-0136-4 (2020).

22 Gimenez-Gomez, P., Fernandez-Sanchez, C. & Baldi, A. J. A. O. Microfluidic Modules with Integrated Solid-State Sensors for Reconfigurable Miniaturized Analysis Systems. **4**, 6192-6198 (2019).

23 Maillard, D., De Pastina, A., Larsen, T. & Villanueva, L. G. Modular interface and experimental setup for in-vacuum operation of microfluidic devices. *Rev Sci Instrum* **90**, 045006, doi:10.1063/1.5088946 (2019).

24 Geng, Z., Gu, Y., Li, S., Lin, B. & Liu, P. A Fully Integrated In Vitro Diagnostic Microsystem for Pathogen Detection Developed Using a "3D Extensible" Microfluidic Design Paradigm. *Micromachines (Basel)* **10**, doi:10.3390/mi10120873 (2019).

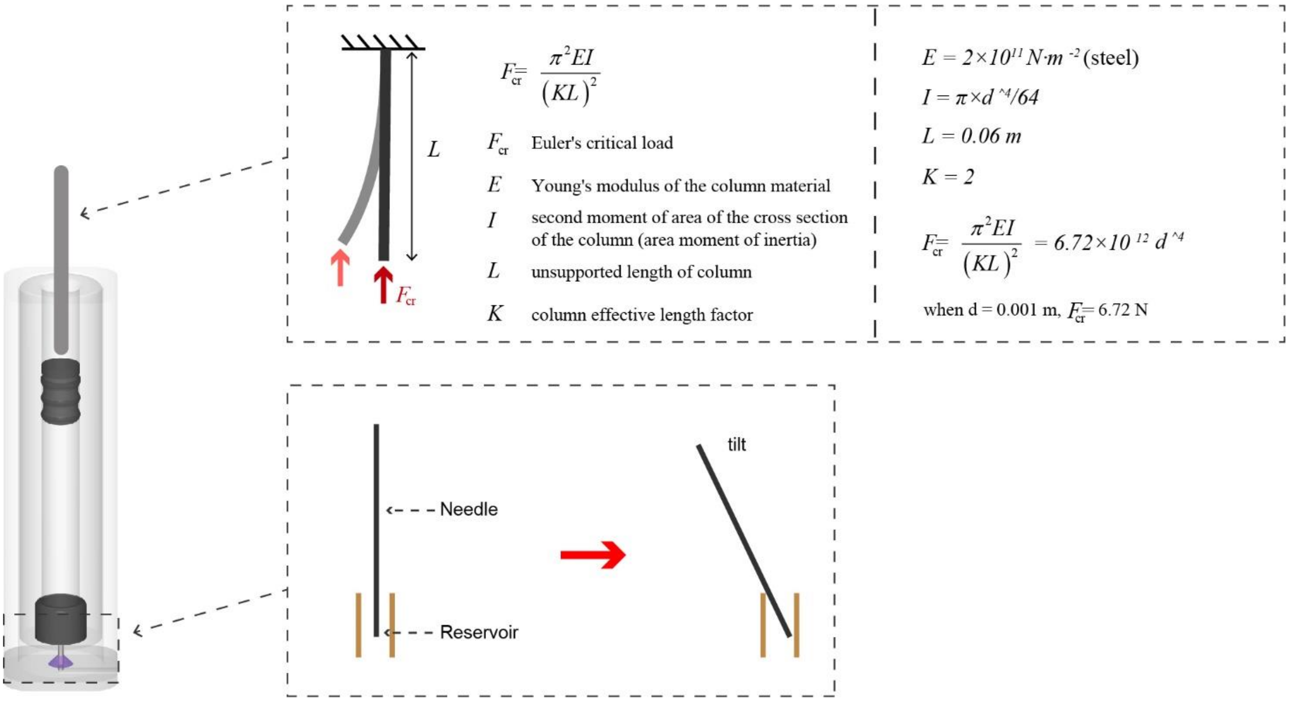

Figure. S1 Key factors affecting barrel diameter.

The factors affecting the barrel diameter can be broadly categorized into two types. Firstly, machining-induced errors play a crucial role. To ensure the sealing integrity of the components, the inner diameter of the barrel must not exhibit significant variations. Theoretical calculations indicate that when the plunger is made of steel and has a diameter of 1mm, it can provide a force of 6.72N without deformation during the downward movement, exceeding the force required in our tests when the barrel diameter is 7mm. Theoretically, the minimum barrel diameter can be below 1mm. However, practical challenges arise during production processes, and maintaining consistent barrel diameters, especially in smaller sizes, is difficult, whether through machining or injection molding (due to the larger aspect ratio).

The second type of influencing factor is errors introduced during assembly and operation. In securing the hollow needle in the reservoir, a certain gap is intentionally left between the reservoir and the hollow needle for ease of assembly. This gap may result in needle tilting, necessitating a proportionate increase in the barrel diameter to accommodate these errors. Additionally, errors are inevitable during the alignment of the plunger with the piston, making it challenging to ensure 100% concentricity. Therefore, a reasonable increase in the barrel diameter is necessary. Taking all these factors into consideration, we have opted for a barrel with an inner diameter of 3mm.

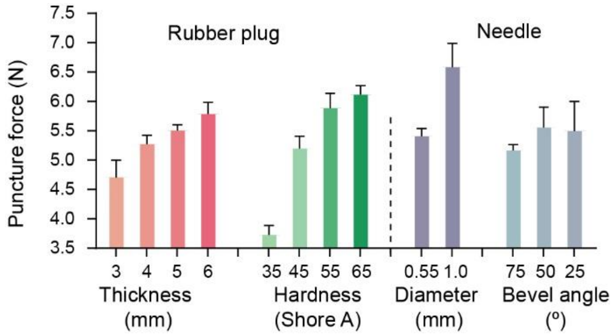

Figure. S2 Effect of rubber plug and hollow needle parameters on penetration force. In the rubber plug thickness test, a consistent hardness of 45° was maintained, whereas in the hardness test, a thickness of 3mm was held constant. The diameters test involved keeping a needle bevel angle of 45° constant, while a diameter of 0.55mm was maintained in the bevel angle test. Error bars represent mean ± s.d. (n = 3).

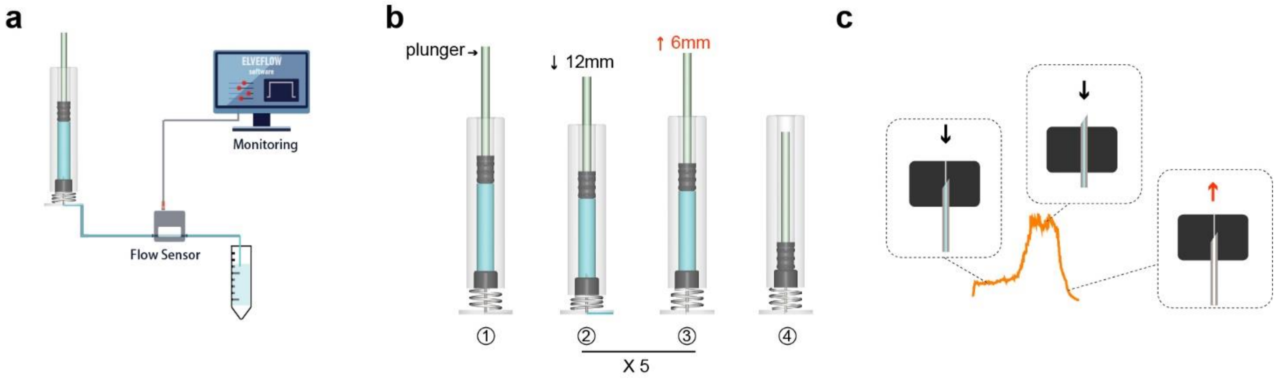

Figure. S3 Experimental details of the on-off cycle for reagent injection. a, Connection of the hollow needle's base to the flow sensor (procured from *Elveflow*) enables the measurement of the flow rate for each release of reagent. **b,** Details of the plunger movement throughout the process involve a downward motion of 12mm, followed by an upward movement of 6mm. This cycle repeats five times until all reagents are completely dispensed. **c,** In each cycle, the position relationship between the hollow needle and the rubber plug corresponds to fluid flow rates. As the plunger drives the piston downward, subtle cracks in the plug, resulting from the previous penetration by the hollow needle, allow fluid to exit the container at a relatively low flow rate due to internal pressure. Once the hollow needle fully pierces the plug, the flow rate reaches its maximum. As the plunger moves upward, relieving pressure, the container reseals.

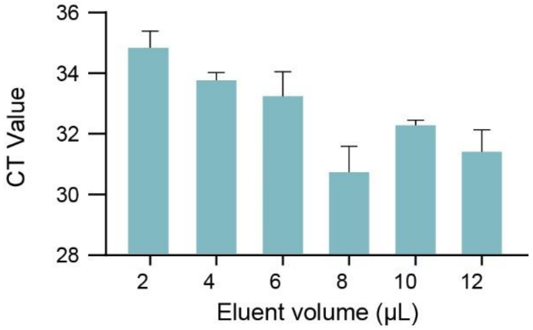

Figure. S4 Optimization of eluted solution volume. The experimental reaction volume is 25 μL, with the addition of 1 μL of elution fluid in the reaction system. Error bars represent mean ± s.d. (n = 3).

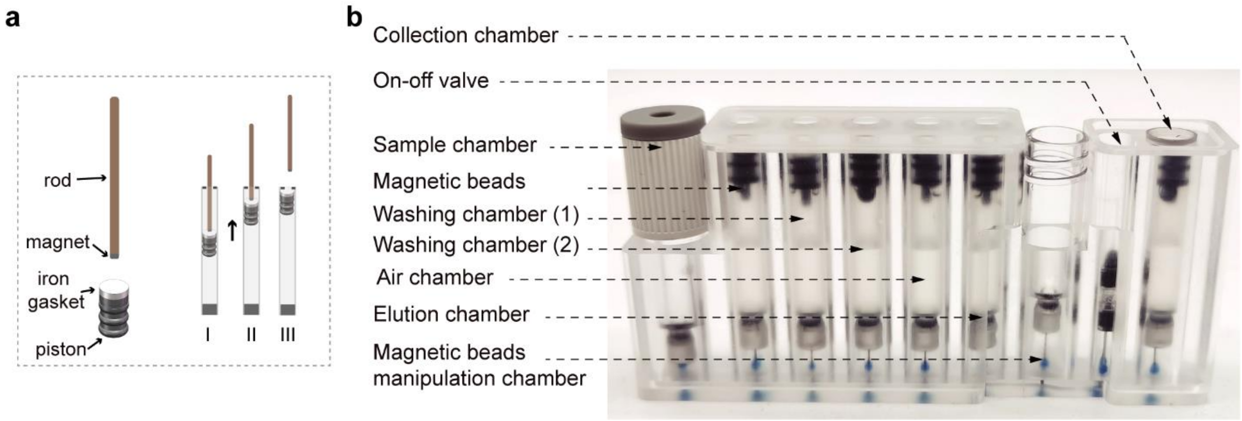

Figure. S5 Structure and operation of the nucleic acid extraction cassette. a, Schematic and operational details of the interaction between the push rod and the rubber stopper. A magnet is fixed at the lower part of the push rod, and an iron gasket is secured at the top of the piston. When the push rod and piston make contact, pulling the push rod upwards causes the piston to move upward due to magnetic force. Once the piston reaches the top, it separates from the push rod as the lid of the cassette catches the piston. b, Photograph and schematic diagram of the microfluidic cassette.

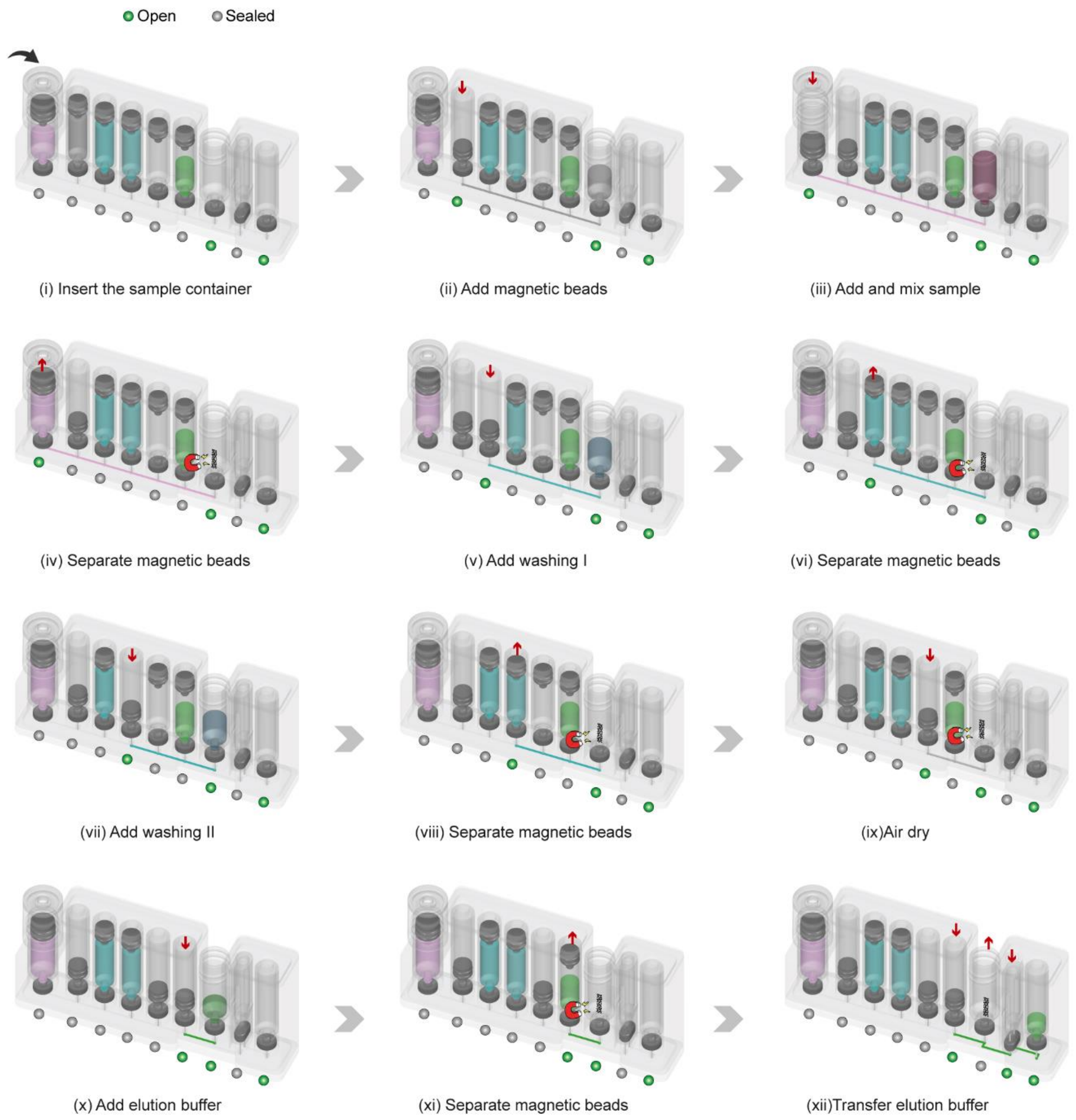

Figure. S6 Fluid schematic of the cassette. (ii-iv) Mixing of the sample with magnetic beads for nucleic acid binding; (v-vi) First washing step; (vii-viii) Second washing step; (ix) Drying of the magnetic beads; (x-xii) Elution of nucleic acids from the magnetic beads.

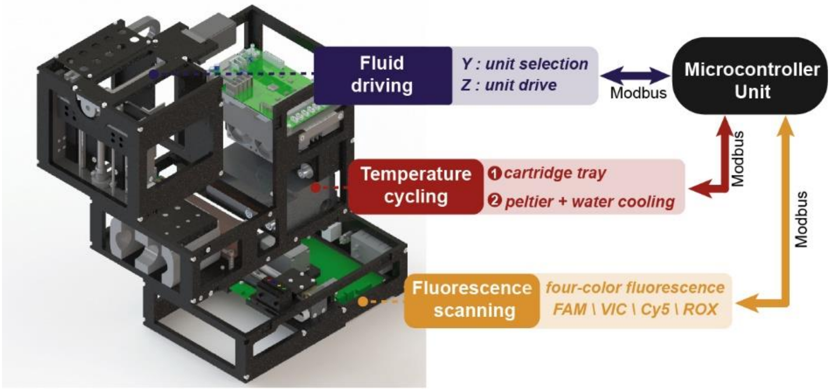

Figure. S7 The modular design of the nucleic acid analyzer. The analyzer is divided into three modules: Fluid Driving, Temperature Cycling, and Fluorescence Scanning. These modules are interconnected to the microcontroller unit through the Modbus protocol.

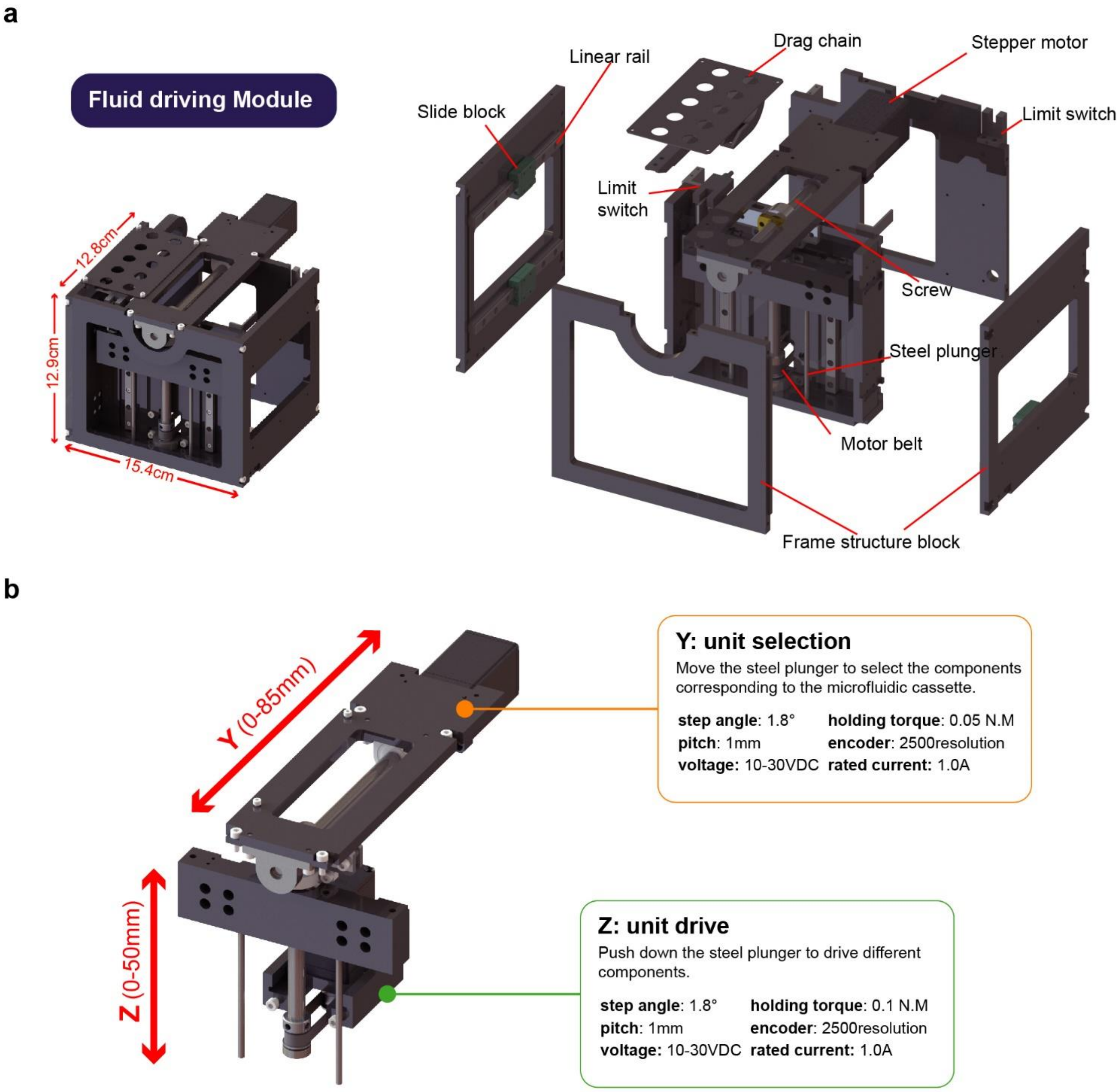

Figure. S8 Schematic and performance parameters of the fluid driving module. a, Exploded view of the fluid driving module. **b,** Functional and performance parameters of the Y and Z axes of the fluid driving module.

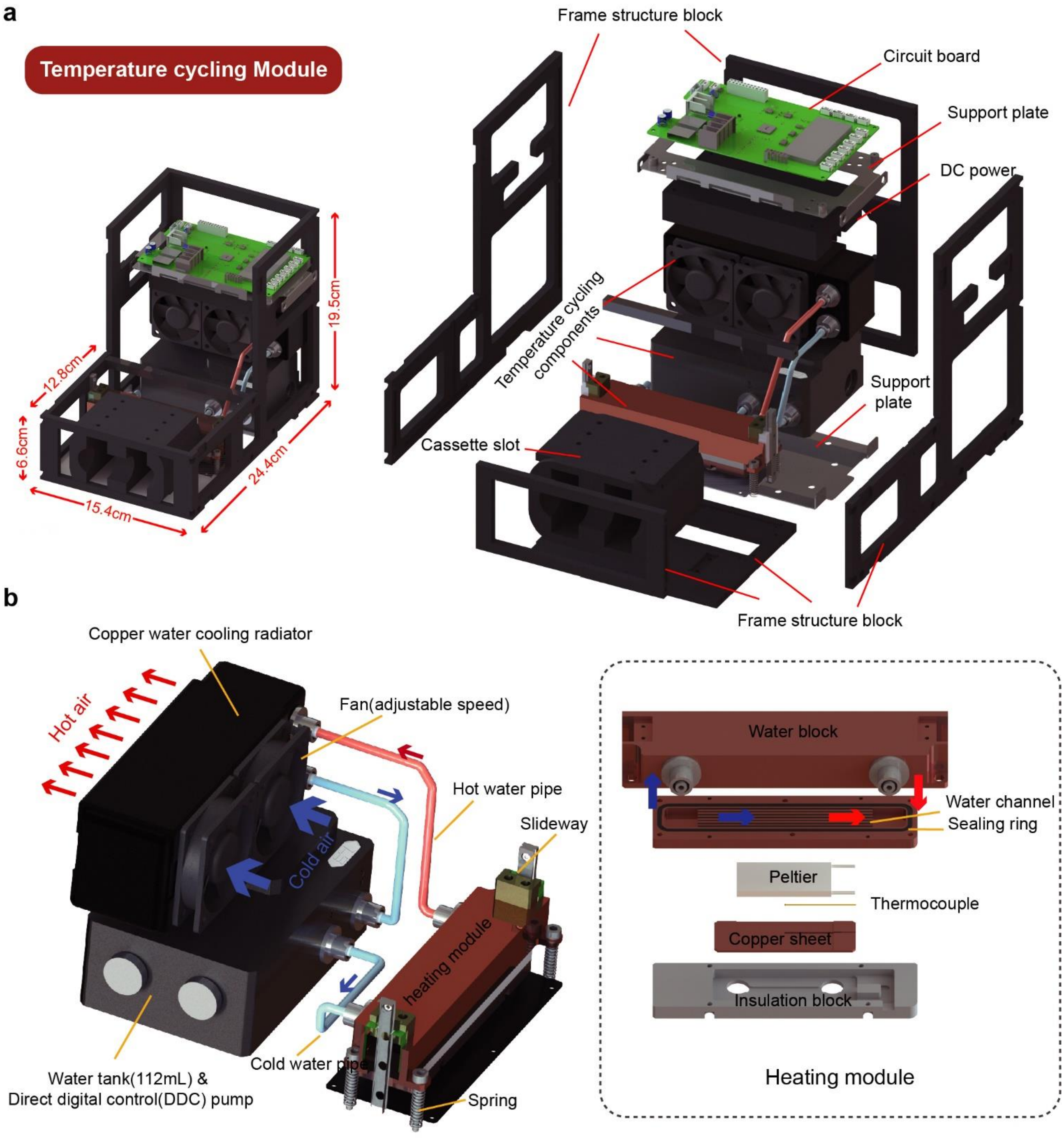

Figure. S9 Schematic and performance parameters of the temperature cycling module. a, Exploded view of the temperature cycling module. **b,** Working principle of the temperature cycling module. The module uses Peltier elements for heating and cooling, and a water-cooling system for heat dissipation.

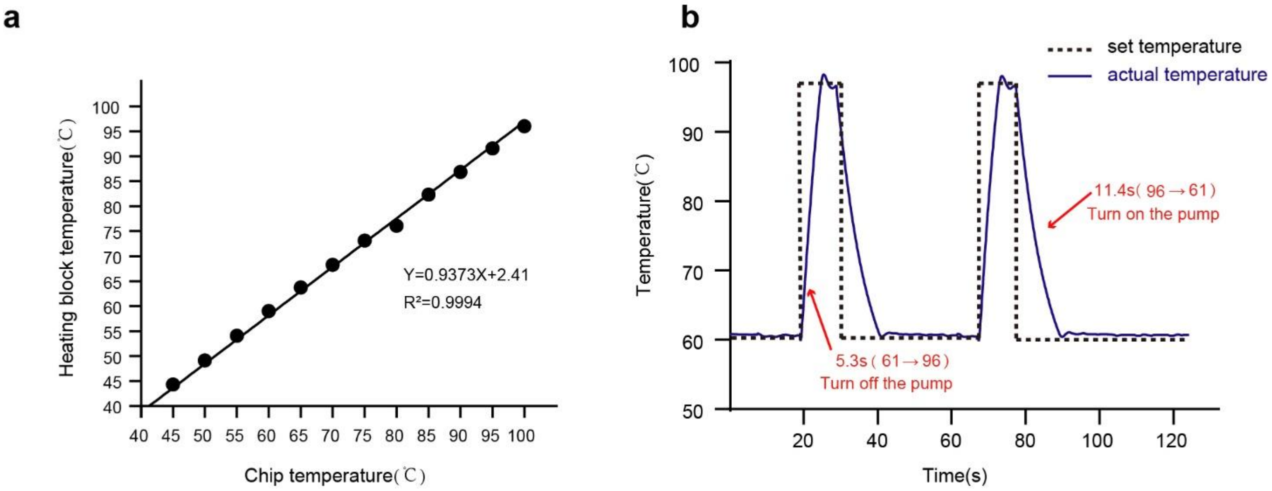

Figure. S10 The performance validation of the temperature cycling module. a, Relationship between the temperature inside the chip and the temperature of the heating block. **b,** Temperature curve inside the chip during the PCR temperature cycling process.

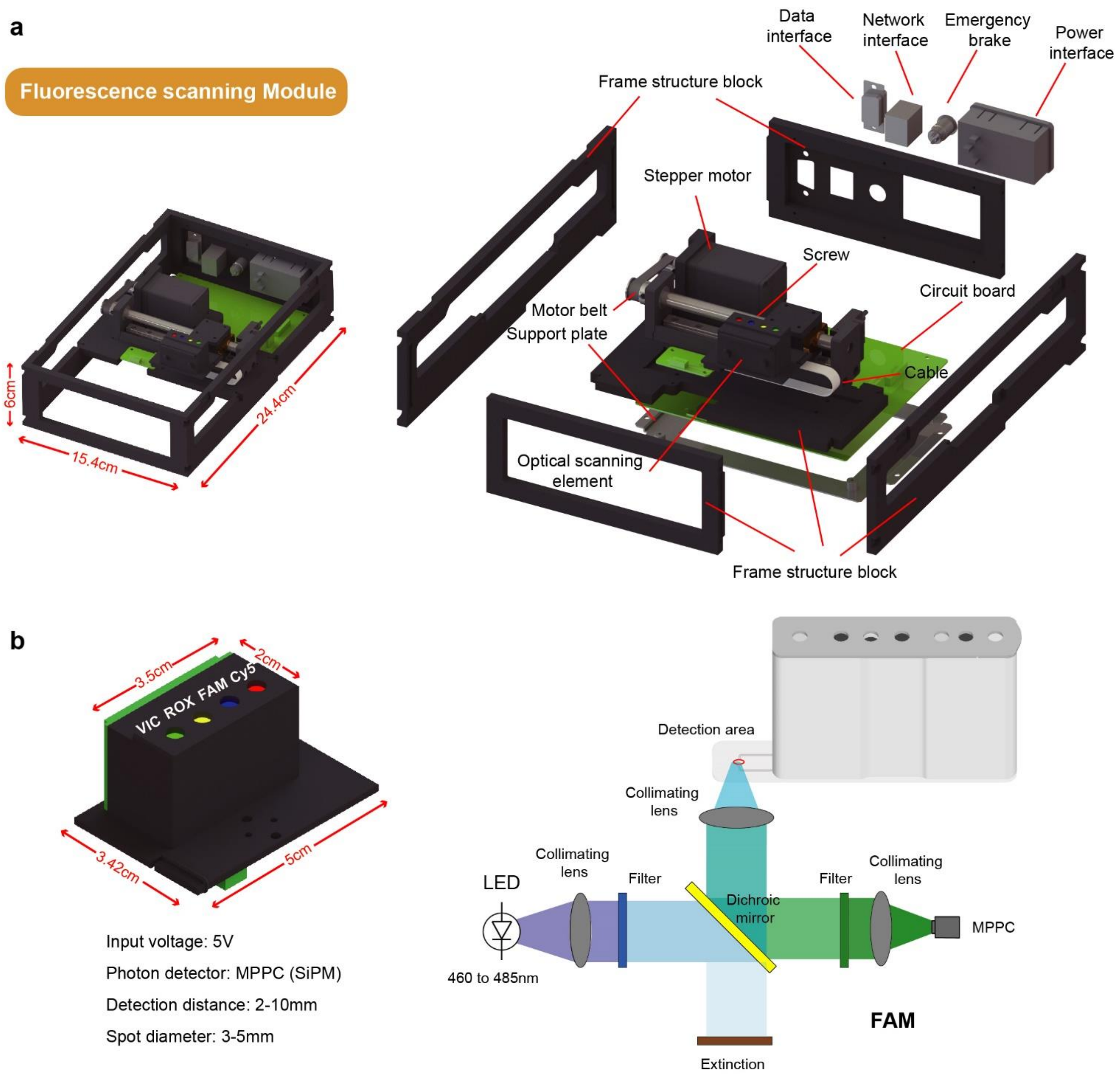

Figure. S11 Structure and performance of the fluorescence scanning module. a, Exploded view of the fluorescence scanning module. **b,** Working principle and detection performance of the fluorescence scanning module.

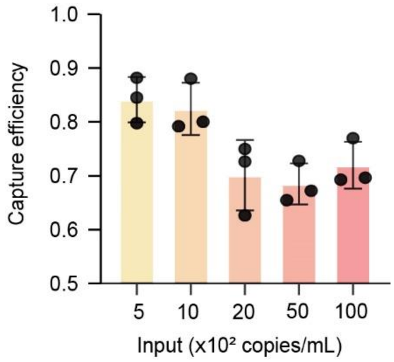

Figure. S12 The RNA capture efficiency of the silica membrane. The membrane is fixed within the cassette chamber, and the sample is laterally passed through it. Various quantities of SARS-CoV-2 RNA templates were introduced to the membrane. The elution solution is used for RT-PCR amplification. Error bars represent mean ± s.d. (n = 3).

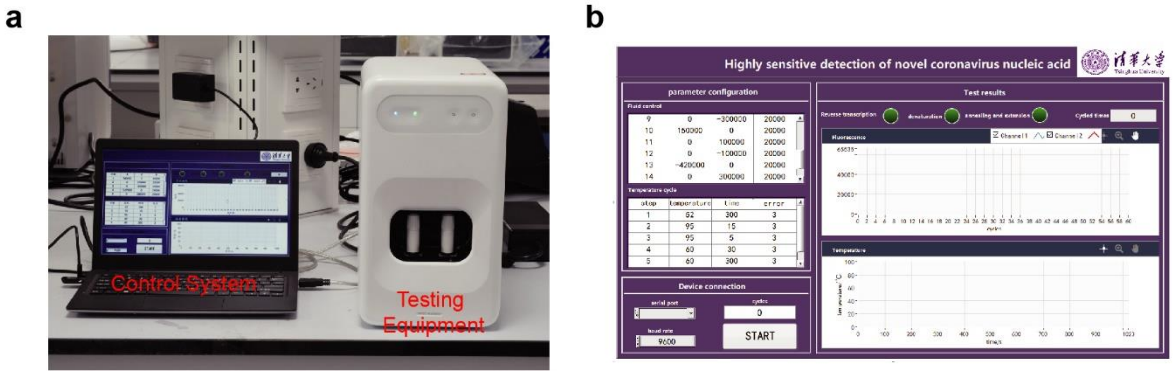

Figure. S13 The iDEP system. a, Composition of the fully integrated analyzer. **b,** LabVIEW control interface. The LabVIEW software interface is designed with parameter configuration, equipment connection, and result display sections.

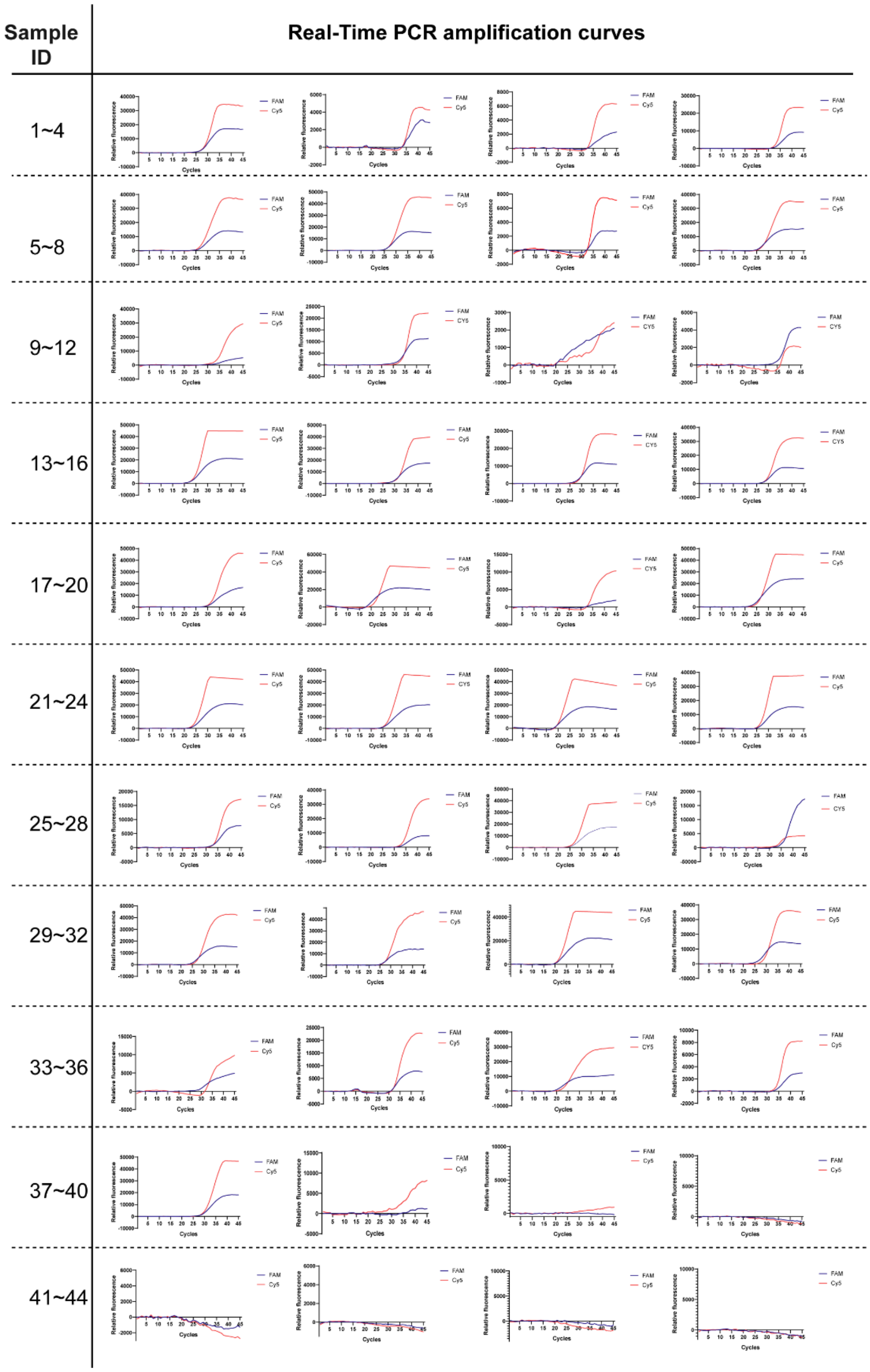

Figure. S14. Fluorescence curve plots from the iDEP system amplification process (1-44).

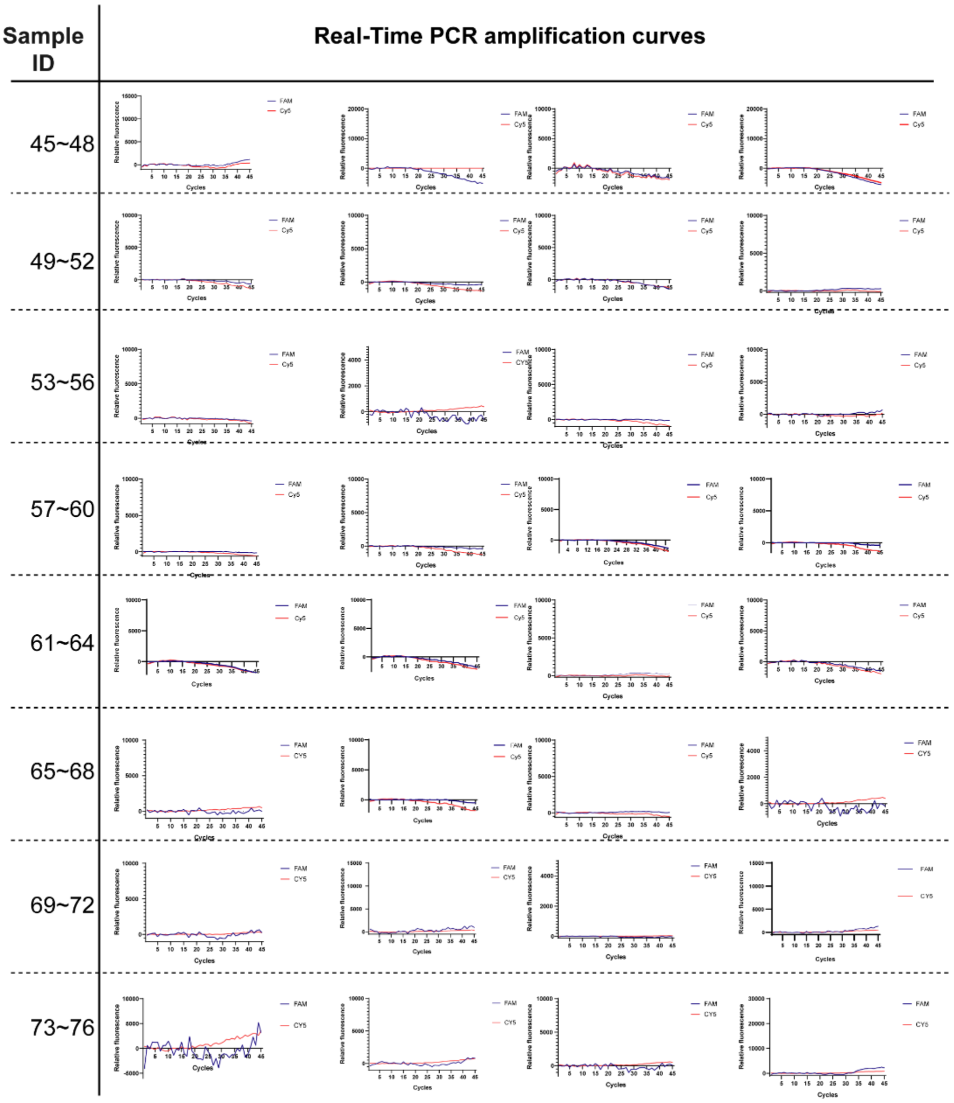

Figure. S15. Fluorescence curve plots from the iDEP system amplification process (45-76).

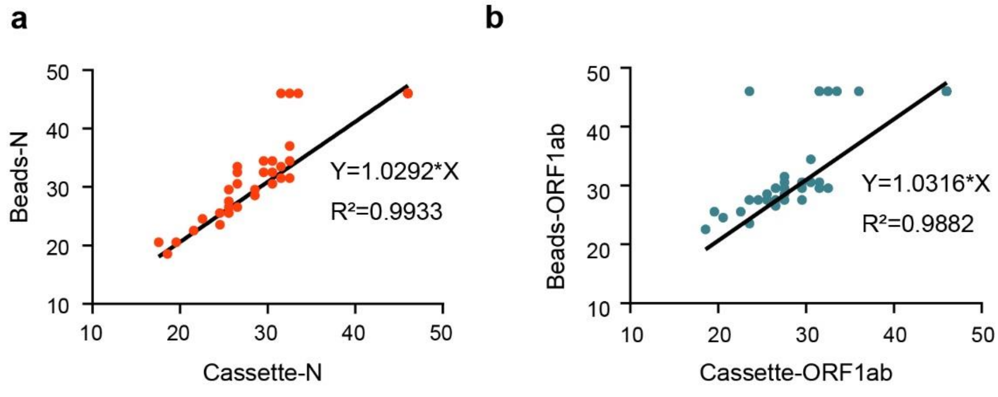

Figure. S16 Comparison of CT values obtained through iDEP and conventional qPCR method. a, Comparative analysis of CT values for the N gene detected using two distinct techniques. **b,** Comparative analysis of CT values for the ORF1ab detected using two distinct techniques.

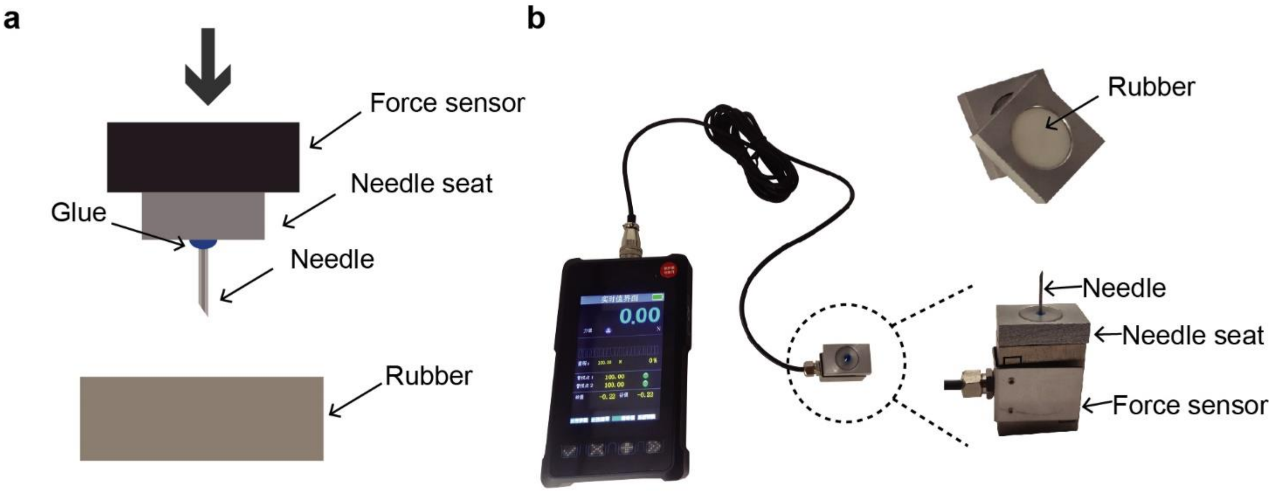

Figure. S17 Force testing apparatus for puncturing rubber with a needle. a, Schematic diagram of force testing for puncturing rubber. **b,** The photograph of the force sensor and a schematic diagram of the fixation of the hollow needle and rubber.

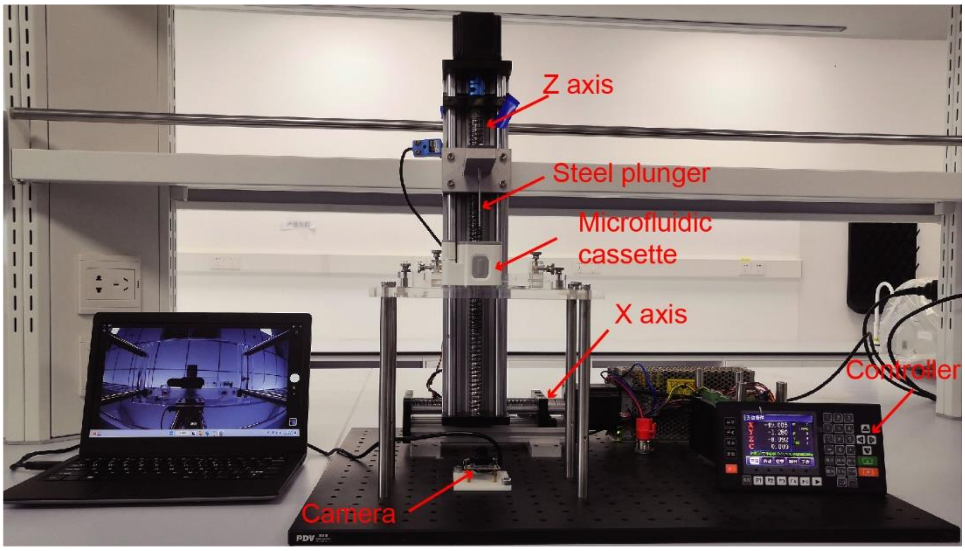

Figure. S18 Photograph of the fluid testing platform. The platform includes motion components in two directions, a motor controller, an image acquisition camera, and a laptop computer for display.
